# Multiscale dispersion-state characterization of nanocomposites using optical coherence tomography

**DOI:** 10.1038/srep31733

**Published:** 2016-08-25

**Authors:** Simon Schneider, Florian Eppler, Marco Weber, Ganiu Olowojoba, Patrick Weiss, Christof Hübner, Irma Mikonsaari, Wolfgang Freude, Christian Koos

**Affiliations:** 1 Institute of Photonics and Quantum Electronics (IPQ), Karlsruhe Institute of Technology (KIT), 76131 Karlsruhe, Germany; 2 Fraunhofer Institute for Chemical Technology (ICT), 76327 Pfinztal, Germany; 3 Institute of Microstructure Technology (IMT), Karlsruhe Institute of Technology (KIT), 76131 Karlsruhe, German

## Abstract

Nanocomposite materials represent a success story of nanotechnology. However, development of nanomaterial fabrication still suffers from the lack of adequate analysis tools. In particular, achieving and maintaining well-dispersed particle distributions is a key challenge, both in material development and industrial production. Conventional methods like optical or electron microscopy need laborious, costly sample preparation and do not permit fast extraction of nanoscale structural information from statistically relevant sample volumes. Here we show that optical coherence tomography (OCT) represents a versatile tool for nanomaterial characterization, both in a laboratory and in a production environment. The technique does not require sample preparation and is applicable to a wide range of solid and liquid material systems. Large particle agglomerates can be directly found by OCT imaging, whereas dispersed nanoparticles are detected by model-based analysis of depth-dependent backscattering. Using a model system of polystyrene nanoparticles, we demonstrate nanoparticle sizing with high accuracy. We further prove the viability of the approach by characterizing highly relevant material systems based on nanoclays or carbon nanotubes. The technique is perfectly suited for in-line metrology in a production environment, which is demonstrated using a state-of-the-art compounding extruder. These experiments represent the first demonstration of multiscale nanomaterial characterization using OCT.

Nanomaterials represent an emerging multi-billion dollar market driven by a vast variety of applications that range from mechanical and civil engineering to energy storage and life sciences. Examples comprise nanocomposite polymers with enhanced mechanical or electronic properties^1^,^2^, functional coatings^3^, flame-retardant materials^4^, advanced drug-delivery systems^5^, and anode materials for Li-ion batteries^6^. These applications mostly rely on the unique properties of nanosize particles, namely huge surface-to-volume ratios, enhanced tensile strengths and superior electrical conductivities as shown by carbon nanotubes (CNT) or other nanofibres^7^, or outstanding barrier properties of nanoplatelets^8^. Properties of nanocomposites depend not only on the size and shape of the particles but also on their dispersion state, characterized by the degree of agglomeration when immersed into a liquid or solid host material. The dispersion state is governed by the nanoparticle properties, by the composition and the physical parameters of the host material, as well as by the processing route adopted for dispersing the nanoparticles in the host material. In order to ensure consistent product quality, the dispersion state must be continuously monitored during fabrication, which has been identified a key challenge both in nanomaterial development and industrial production^9^. In solid media, dispersion-state characterization mainly relies on microscopic imaging. For small nanoparticles, this requires high-resolution techniques such as scanning-electron microscopy (SEM) of specially prepared sample surfaces^10^, or transmission electron microscopy (TEM) of microtome slices^11^. In both cases, sample preparation is laborious and costly and not well suited for quality control or process development, where sample processing and analysis have to be continuously iterated. Moreover, SEM and TEM are limited to small sample volumes, which are not necessarily representative of the entire batch. Light microscopy, on the other hand, can reduce the experimental effort^12^, but is limited to the identification of large agglomerates in sufficiently transparent samples and areas close to the surface. In contrast to image-based methods, light scattering techniques have proven to be viable tools for measuring particle size distributions from large sample volumes. Static light scattering (SLS) relies on angle-resolved and/or spectrally resolved detection of scattered light^13^,^14^ and is based on a rather complex optical setup, especially when large scattering angles have to be taken into account. Moreover, for solid samples, it is challenging to separate scattering inside the sample volume from contributions of the rough sample surface. Dynamic light scattering (DLS) methods exploit temporal fluctuations of interference patterns of scattered light to calculate the Brownian motion and the hydrodynamic diameter of particles within the respective solvent^15^. This technique is limited to low concentrations, and can only be applied to liquid media. In addition, both SLS and DLS suffer from limitations in dealing with mixtures of particles having vastly different diameters that may range, e.g., from a few nanometres to hundreds of micrometres, as is often the case for nanocomposites with poorly dispersed nanoparticles. Partial wave spectroscopic microscopy (PWS)^16^ has been used for investigation of nanoscale refractive index fluctuations, which can be an early indicator of carcinogenesis, yet without providing a link to the size of the scatterers. X-ray diffraction (XRD)^17^ or small-angle X-ray scattering (SAXS)^18^ finally rely on diffraction or scattering of X-rays in the sample and are able to reveal the atomic or molecular arrangement inside the material. However, high instrumental effort and a small probing region limit the application range of these methods to the laboratory environment. Hence, none of the aforementioned techniques can meet the stringent requirements associated with industrial process development and quality control, which comprise robustness of the measurement method, fast analysis, the capability to characterize representative sample volumes without laborious sample preparation, good mechanical and thermal robustness of the measurement system, and the possibility to integrate the measurement system into the processing line for enabling in-line process control. The lack of adequate analysis methods for dispersion-state characterization is considered one of the major obstacles towards large-scale industrial processing and exploitation of nanomaterials.

As an alternative, optical coherence tomography (OCT)^19^,^20^,^21^ was proposed as a tool for nanocomposite characterization^22^,^23^. OCT provides three-dimensional imaging data from the bulk of the sample and avoids expensive sample preparation. However, previous demonstrations have been limited to image-based analysis of composites containing rather large microparticles combined with wavelet-based processing of the image data^23^. Due to the limited resolution of OCT imaging, this method cannot provide information about the nanoscopic structure of the nanocomposite such as the particle size. A further method uses a spectral-domain OCT setup and measures the size of particles suspended in a fluid by the temporal variation of the optical phase due to particle diffusion^24^,^25^. Particle sizes range from 15 nm to 625 nm. The main disadvantage is that only particles suspended in a fluid can be investigated, and that parameters like viscosity and temperature need to be tightly controlled. Another technique is angle-resolved OCT^26^, where the angular dependence of the scattered intensity is evaluated according to Mie’s theory. However, the angular range is limited to about 0.5 rad due to practical reasons, and the particle size which can be detected is not smaller than 5 μm in diameter. A further approach is low-coherence spectroscopy, which allows for the extraction of wavelength-dependent scattering coefficients of the investigated samples and compares well with Mie scattering calculations^27^. The aforementioned techniques serve well for the determination of scattering parameters from nanoparticle samples, but miss the multiscalar approach, including imaging of large agglomerations. A technique using a setup similar to spectral-domain OCT is super-resolution imaging relying on spectral encoding of spatial frequency (SESF)^28^. With that approach, sub-micrometre imaging has been demonstrated, but exact nanoparticle sizes cannot be extracted due to the still limited resolution. In summary, OCT-based quantitative and qualitative characterization of composites at the nanoscale still remains to be shown.

In this paper we demonstrate that OCT represents an attractive tool for fast and robust dispersion-state characterization of composite materials over a wide range of particle and agglomerate sizes both on the micrometre and on the nanometre scale. The technique pursues a multiscale approach: Using a theoretical model of light scattering in the sample, we accurately measure particle sizes down to 140 nm. Particle agglomerates with sizes of up to hundreds of micrometres can be easily detected by applying dedicated image processing techniques to the OCT data. Both methods can be performed *in situ*, without prior sample preparation, in both liquid and solid materials, and are applicable to laboratory investigations as well as to in-line process control in industrial production. Using a model system of polystyrene nanoparticles dispersed in water, we prove the reliability and accuracy of our sizing technique. We further apply the technique to an epoxy resin filled with multi-wall carbon nanotubes (MWCNT). The results of OCT-based scattering analysis show good correlation with independently measured material properties, thereby outperforming conventional characterization techniques based on light microscopy. Finally, we show that our technique is also perfectly suited for in-line metrology in a production environment. To this end, we integrate our system with a state-of-the-art industrial compounding extruder using a dedicated optical probe. The OCT system operates reliably during the compounding process and allows to immediately examine the impact of extruding parameters on the dispersion state of the material. We believe that OCT will pave the path towards industrially viable nanomaterial characterization and process control.

## Materials and Methods

### Swept-source optical coherence tomography (OCT) system

Optical coherence tomography (OCT) evolved greatly in the past decades. The technique provides microscopic resolution in volumetric imaging and highly sensitive detection of backscattered optical power. OCT opened a wide field of applications reaching from ophthalmology in medical diagnostics^29^ to particle and defect characterization in material sciences^22^,^30^. Among various implementations, swept-source OCT (SS-OCT) offers a particularly attractive combination of highest sensitivity and high imaging speed^21^.

**Figure 1:**
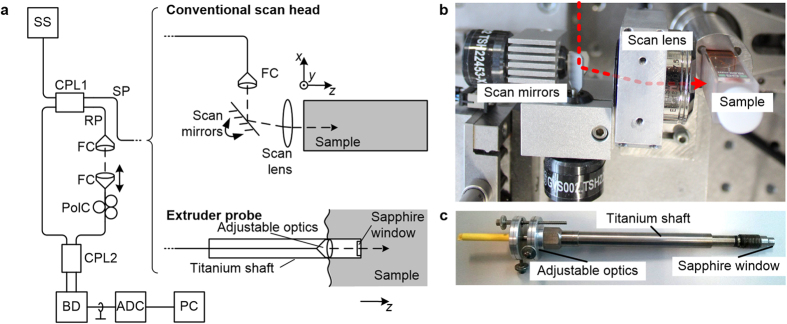
Fibre-based swept-source optical coherence tomography (SS-OCT) setup for laboratory measurements and in-line process control. (**a**) Schematic setup of the SS-OCT. SS: Swept-wavelength source, scanning range 1260–1370 nm; CPL1,2: fibre-based directional coupler; SP: sample path; RP: reference path; FC: fibre collimator; PolC: polarization controller; BD: balanced photodetector; ADC: analogue-to-digital converter; PC: personal computer. (**b**) Laboratory scan head with galvo-based scanners and a scan lens for 3D-imaging. The red line indicates the light beam towards the sample. (**c**) Extruder probe for in-line measurements. The probe consists of a titanium shaft for a high temperature environment and comprises a sapphire window towards the sample.

The SS-OCT setup used in this work is depicted in Fig. 1(a). In general, an OCT system measures the position and the strength of a multitude of scatterers along a light path in a sample. To this end, the electric field that is backreflected from a sample is compared in amplitude and phase to a reference field. Both the sample and the reference field are derived from the same optical swept-wavelength source (SS). In our experiments, we use an SS laser with central wavelength of 1315 nm and a wavelength scanning range of 1260–1370 nm, a scan rate of 1 kHz, and 10 dBm average output power (model s3, Micron Optics Inc., Atlanta, GA, USA). The scan range of the laser corresponds to a theoretical depth resolution of 7 μm, which compares well to the experimentally observed resolution of 11 μm. A first fibre-based directional coupler (CPL1, splitting ratio 50:50) is used to split the power among the sample path (SP) and the reference path (RP). The reference path contains a free-space section allowing for precise matching of the RP and SP length. Backscattered light from the sample and light travelling along the RP is superimposed in a second fibre-based coupler (CPL2, splitting ratio 50:50) and coupled to a balanced photodetector (BD, model PDB430C, Thorlabs, Munich, Germany). The output current of the BD contains patterns resulting from interference of the backscattered field with the reference field. The BD suppresses intensity noise from the strong RP signal and enables a large dynamic range and a high sensitivity, defined by the lowest detectable backscatter from the sample, of −110 dB. The electrical signal is digitized by a 16 bit analogue-to-digital converter (ADC, model ATS660, Alazar Technologies Inc., Pointe-Claire, Canada) and processed in a personal computer (PC). The amplitude and the position of the backscatter along the light path can be obtained by Fourier analysis of the photocurrent as a function of optical frequency^21^. One depth-scan (A-scan) consists of 768 measurement points with 8 μm step size. We extract the backscatter strength, which denotes the ratio of backscattered optical power to optical power incident on the sample.

In the course of this work, the SP of the SS-OCT system is connected to two different scan heads: First, to a conventional OCT scan head for offline characterization of laboratory samples, Fig. 1(b), and second, to a specially developed probe head for in-line dispersion characterization during the compounding process in an industrial extruder, Fig. 1(c). This probe is designed for the harsh environmental conditions at a nanocomposite production line and must tolerate vibrations, high temperatures of 250 °C, and high pressures of 200 bar. The probe features a titanium shaft and a sapphire window towards the sample, and is designed to allow for adjustment of focal length and the axial position of the focus within the sample.

### Model-based dispersion state analysis and sizing of nanoparticles

Big agglomerates of nanoparticles with dimensions larger than the resolution limit of the OCT system can be detected directly by imaging. However, size information on nanoscale agglomerates and single particles is relevant as well. In this section we show that model-based analysis of OCT backscatter measurements allows to extract scattering parameters that are correlated with the dispersion state of the material so that even small particle sizes can be determined.

**Figure 2:**
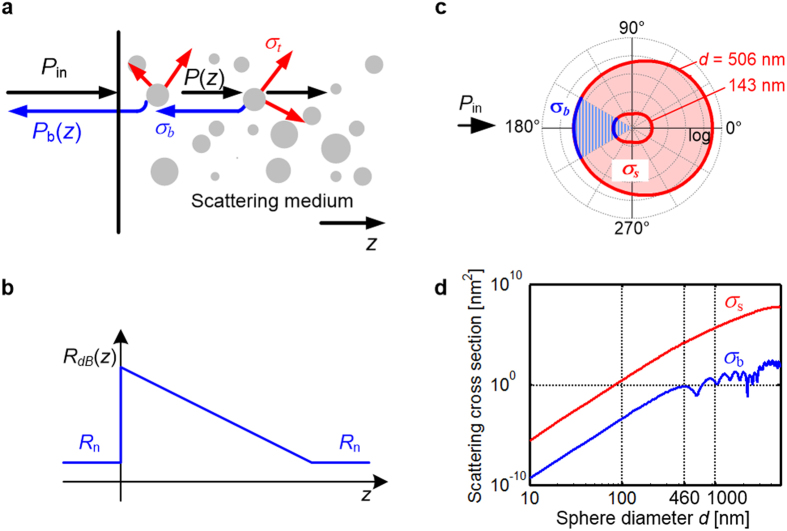
Concept of model-based dispersion state analysis and sizing of nanoparticles. (**a**) Single scattering model: Incident light is scattered at maximum once inside the medium. A light beam with power *P*_in_ enters the sample. At each particle (grey), a first portion (*σ*_b_, blue) of the incident light in the respective depth *z* is scattered back into the numerical aperture of the optical system, a second portion (*σ*_t_, red) is scattered into all other directions or is absorbed, and the remaining third portion (black) propagates deeper into the medium. (**b**) Schematic profile of the logarithmic backscattering factor *R*_dB_(*z*) = 10 lg(*R*(*z*)). (**c**) Direction-dependent scattering lobes according to Mie’s theory, plotted here for the example of polystyrene (PS) nanospheres (diameters 506 nm and 143 nm, refractive index *n*_PS_ = 1.57 at 1315 nm) in aqueous dispersion (*n*_H20_ = 1.33). *σ*_s_: total scattering (red); *σ*_b_: backward scattering (blue). (**d**) Total scattering *σ*_s_ and backscattering *σ*_b_ cross sections for non-absorbing polystyrene spheres in aqueous dispersion.

The analysis relies on a single-scattering model assuming that incident light is scattered at maximum once within the medium, similar to the approach used by Kodach *et al*.^31^. This model is found to be appropriate for weakly scattering samples and for an analysis of moderate scattering depths^32^. As depicted in Fig. 2(a), a light beam with input power *P*_in_ enters the sample. At each particle (grey), a first portion (blue) of the incident light in the respective depth *z* is scattered back into the numerical aperture of the optical system, a second portion (red) is scattered into all other directions or is absorbed, and the remaining third portion (black) is propagating deeper into the medium. The total scattering *σ*_s_ and absorption cross section *σ*_a_ can be described by the extinction cross section *σ*_t_ = *σ*_s_ + *σ*_a_ of a single particle, or by the extinction coefficient *μ*_t_ = *Nσ*_t_ for an ensemble of particles with volume number density *N*. In analogy, the backscatter is described by the backscatter cross section *σ*_b_, or by the backscatter coefficient *μ*_b_ = *Nσ*_b_ of the material.

The depth-dependent decay d*P(z)*/d*z* = −*μ*_t_*P*(*z*) of the forward-travelling power *P*(*z*) is dictated by the extinction coefficient *μ*_t_, leading to an exponential decay of optical power *P*(*z*) = *P*_in_*e*^−*μ*^_t_^z^ inside the sample. For a given depth *z*, the power in a depth element *δz* as scattered back into the numerical aperture of the optical system amounts to *P*_b_(*z*) = *μ*_b_*δzP*(*z*)*e*^−*μ*^_t_^*z*^, where *e*^−*μ*^_t_^*z*^ accounts for the extinction of the backscattered light during backpropagation. The signal measured in an OCT scan corresponds to the depth-dependent backscatter *P*_b_(*z*) = /*P*_in_. In the presence of a depth-independent noise floor *R*_n_, the depth-dependent backscatter signal measured by the OCT system is




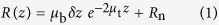




A semi-logarithmic plot *R*_dB_(*z*) = 10 lg(*R*(*z*)) of this backscatter signal is depicted in Fig. 2(b). The background noise term *R*_n_ defines the sensitivity limit of the OCT system. For real OCT systems, this background noise is sometimes dominated by relative intensity noise (RIN) of the swept source, which may lead to a depth-dependent noise floor. In contrast to that, the noise floor in our system originates from thermal noise of the receiver electronics, which exhibits a white power spectrum and is hence constant over the depth range of interest. Note that all measurements for particle sizing are taken under oblique incidence of the OCT beam on the sample surface. This avoids occurrence of isolated reflection peaks at the sample surface such that the signal model according to Eq. (1) can be directly used to fit the measurement data.

In real OCT measurements, the measured backscatter depends on further parameters, which need to be determined in a calibration step. This comprises the divergence of the measurement beam, the decay of the coherence function of the swept laser, and the absorption of the matrix material which surrounds the scatterers. These influences are approximated by including two calibration factors^22^,^33^ *q* and *Q* in the single-scattering model according to Eq. (1),




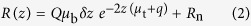




Both calibration factors are determined by comparing measured backscatter of a NIST-traceable polystyrene particle standard (246 nm diameter polystyrene nanospheres in 0.5 wt.% aqueous dispersion, BS-Partikel GmbH, Wiesbaden, Germany) with the model calculations according to Eq. (1).

In the special case of spherical scatterers with a size in the order of the wavelength, the scattering cross section *σ*_s_ can be modelled by means of Mie’s theory^34^. As an example, Fig. 2(c) shows direction-dependent scattering lobes of water-dispersed polystyrene nanospheres with diameters 143 nm and 506 nm. The larger sphere (506 nm diameter) shows stronger total scattering (red), but less pronounced fractional backscatter as compared to the small sphere (143 nm diameter). For a small aperture of the scan head (1° half-angle, corresponding to a theoretical and experimental lateral resolution of 28 μm and 36 μm, respectively), the Mie scattering lobes can be parameterized using the total scattering cross section *σ*_s_ and the backscattering cross section *σ*_b_. Figure 2(d) shows both cross sections *σ*_s_ and *σ*_b_ as a function of the sphere diameter *d* for polystyrene (PS) spheres (refractive index *n*_PS_ = 1.57) dispersed in water (refractive index 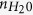 = 1.33) at a wavelength of 1315 nm. In the limit of small diameters *d*, Mie scattering can be approximated by Rayleigh scattering. In this regime, both scattering cross sections increase in proportion to *d*^6^. For larger diameters, the spheres show resonances, which lead to dips in the backscattering cross section *σ*_b_, whereas the total scattering cross section remains unaffected. The relationship between backscattering cross section *σ*_b_ and particle size is unambiguous only if the particle diameter is smaller than 460 nm, which corresponds to roughly half the material wavelength in the polystyrene spheres.

In real measurements, the total scattering cross section *σ*_s_ and the backscattering cross section *σ*_b_ cannot be assessed directly. Instead, only the extinction coefficient *μ*_t_ = *Nσ*_t_ and the backscatter coefficient *μ*_b_ = *σ*_b_ are extracted from the backscatter signal *R*(*z*). For non-absorbing particles in the Rayleigh scattering regime, particle size and concentration cannot be separately evaluated, since both *σ*_s_ and *σ*_b_ increase with *d*^6^, Fig. 2(d). As an example, for particle sizes of less than 150 nm (*λ*/9), we can safely assume Rayleigh scattering, and the backscattering probability *p*_b_ = *σ*_b_/*σ*_s_ stays constant within 10%. As a consequence, a low concentration of bigger particles cannot be distinguished from a high concentration of smaller particles. However, for the case of nanomaterial characterization, the total volume of nanoparticles added to the sample is usually known. For an increasing degree of dispersion, the average size *d* of the agglomerates decreases and their volume number density *N* increases in proportion to *d*^−3^. Together with the *d*^6^-dependence of the scattering cross sections in the Rayleigh regime, this leads to an overall decrease of the scattering coefficients in proportion to *d*^3^, which allows robust separation of particle size *d* and volume number density *N*. Note that this applies to non-absorbing particles only. If the extinction coefficient *μ*_t_ is dominated by the contribution of absorption rather than scattering, an increased degree of dispersion could lead to an increase of *μ*_t_. This is due to the fact that the breaking-up of agglomerates exposes more particles from the inner region to the incident light. These particles from the inner region were formerly shielded from light by the absorbing outer shell, and did therefore not contribute to attenuation. If absorption dominates, the effect of increasing extinction with increasing number of separated absorbing particles can be exploited for the analysis of the dispersion state. We use this approach in the section on dispersion analysis of epoxy-CNT composites.

### Image-based dispersion state analysis

Although nanocomposites ideally feature a homogeneous distribution of nanosized fillers, in practice, microscale agglomerates cannot be completely avoided. The size of the agglomerates could reach several hundreds of micrometres, especially at the beginning of the so-called compounding process, which usually exploits shear forces to break up particle agglomerates into their nanoscale constituents. Therefore, a dispersion-state analysis suitable for process monitoring should be able to cope with nanometre to micrometre sized objects. This section is dedicated to imaging and analysis of agglomerates in the micrometre range.

**Figure 3:**
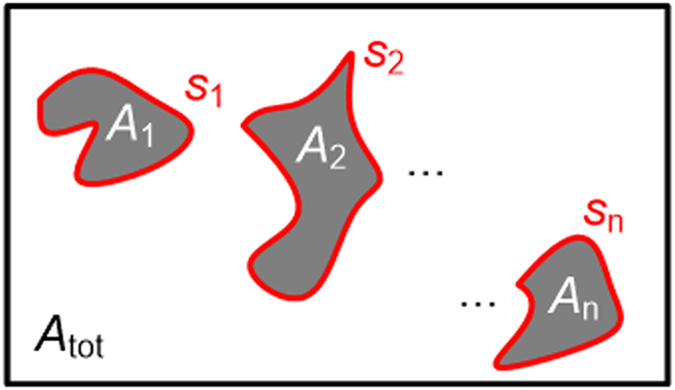
Illustration of agglomerate areas and perimeters in a cross-sectional OCT image. The total image cross section amounts to *A*_tot_; the quantities *A*_1_ … *A*_n_ denote the areas (grey) and the quantities *s*_1_ … *s*_n_ (red) denote the perimeters of the agglomerates. The area fraction (AF) is a measure for total agglomerate content and relates the agglomerate areas *A*_1_ … *A*_n_ to the total image cross section *A*_tot_. The perimeter-to-area ratio (PAR) is a measure for the size of the found agglomerates and relates perimeters *s*_1_ … *s*_n_ to the areas *A*_1_ … *A*_n_.

Optical coherence tomography can be used to visualize agglomerates, if their dimensions exceed the spatial resolution *δx, δy, δz* of the imaging system in *x, y* and *z*-direction (about 10 μm), and if the backscattering is stronger than the background noise. Below these limits, an analysis based on a scattering-model has to be applied. To identify agglomerate regions with stronger backscatter within the OCT image, we use an image segmentation algorithm based on seeded region growing^35^. For quantitative information related to agglomerate size and number, two independent parameters are extracted from the segmented images, namely the area fraction (AF) and the perimeter-to-area ratio (PAR) of the agglomerates, see Fig. 3. The area fraction relates the image area covered by all identified agglomerates with individual area *A*_i_ to the entire imaging cross section *A*_tot_,




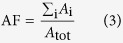




For a given volume fraction of the nanosize filler, and assuming spherical agglomerate shapes which are accurately detected by an ideal measurement system, the AF would be independent of the agglomerate diameter. This can be understood by the following consideration: Assume that each spherical agglomerate splits and decreases in radius by a factor of ν. The total amount of material remains constant, therefore the number of (smaller) agglomerates in the volume increases by ν^3^, whereas the number of agglomerates in the measurement plane increases by ν^2^. The average area *A*_i_ of each individual cross section in the measurement plane decreases in proportion to ν^2^. Therefore the radius change of the agglomerates has no effect on AF. This would render AF as non-indicative for agglomerate analysis. For real measurement systems, however, a decreasing average agglomerate size will increase the number of agglomerates that are smaller than the detection threshold of the image-based analysis technique. In this case, a decrease of AF is observed which is correlated with a decreasing agglomerate size. In our experiments, the detection threshold is set to three times the standard deviation of the background noise floor in the image. While the threshold influences the measured percentage of the area fraction, our choice of the threshold level suffices to judge the dispersion of a certain sample type.

As a further parameter, the perimeter-to-area ratio (PAR) relates the sum of all agglomerate perimeters *s*_i_ to the sum of all agglomerate areas *A*_i_,




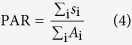




Assuming agglomerates of equally distributed shapes, the PAR depends only on the average size of the agglomerates and is otherwise independent of the number density. A change in the average shape of all agglomerates during the dispersion process would influence the PAR, but this is not to be expected for typical dispersion processes that rely on milling or shear-strain-induced breaking of agglomerates. If the dimensions of each agglomerate decrease by a factor of ν, the perimeter decreases by *v*, while the cross sectional area decreases by *v*^2^. Accordingly, the perimeter-to-area ratio of each agglomerate increases by a factor of ν, and the same applies to the overall PAR, independently of the filler content. Image-based analysis is applied to OCT measurements of nanocomposites both offline and during production.

## Results and Discussion

To prove the viability of OCT as a tool for characterization of nanoparticles and nanocomposite materials, we performed a series of experiments comprising accurate nanoparticle sizing as well as nanocomposite dispersion state analysis in liquid and solid materials. Our characterization employs image evaluation and a simplified model-based scattering analysis. We show that OCT methods are useful for measuring nanoscopic particle sizes as well as for analyzing the dispersion of agglomerates in the micro- and millimetre regime. The technique can even cope with highly absorbing CNT-loaded materials and is well suited for in-line process control.

### Model-based sizing of nanoscale particles

**Figure 4:**
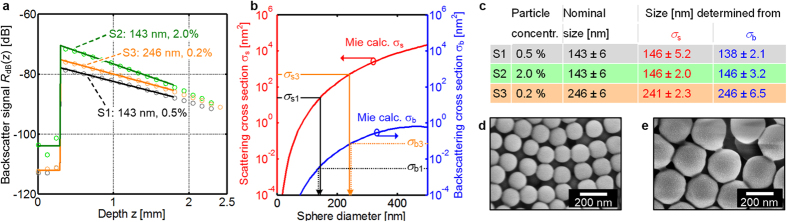
Size determination of sub-wavelength nanoparticles with OCT. (**a**) Measured OCT depth scans of aqueous dispersions of polystyrene (PS) nanospheres (S1: 143 nm diameter, 0.5 wt.%, grey circles: measurement, black line: fit; S2: 143 nm diameter, 2.0 wt.%, light green circles: measurement, green line: fit; S3: 246 nm diameter, 0.2 wt.%, light orange circles: measurement, orange line: fit), fitted with the calibrated single-scattering model according to Eq. (2). (**b**) Total scattering (red) and backscattering (blue) cross sections, according to Mie’s theory for polystyrene spheres in water. Both scattering cross sections are compared with the measurements, depicted only for the samples S1 (*σ*_s1_: total scattering cross section, *σ*_b1_ backscattering cross section) and S3 (*σ*_s3_, *σ*_b3_, respectively). Both parameters can be attributed to sphere diameters, horizontal axis. (**c**) Comparison of nominal particle sizes with measured sizes. Relative (absolute) deviations from the nominal values are maximal 4% (5 nm). (**d,e**) Scanning electron micrograph (SEM) of the nanospheres having diameters of 143 nm and 246, respectively.

Polystyrene (PS) nanospheres (refractive index *n*_PS_ = 1.57) in aqueous dispersion (refractive index 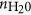 = 1.33) are used as a model system to prove the viability of OCT as a tool for model-based nanoparticle sizing. This model system offers higher refractive index contrast than the sample systems investigated in following sections (polyamide/clay: *n*_PA_ = 1.59, *n*_Clay_ = 1.54; polypropylene/clay: *n*_PP_ = 1.49, *n*_Clay_ = 1.54). All these samples offer sufficient backscattering levels. In this experiment, we characterize dispersed NIST-traceable polystyrene particles (BS-Partikel GmbH, Wiesbaden, Germany) with diameters of 143 nm and 246 nm. The results are depicted in Fig. 4. The particle size is determined by fitting the calibrated single scattering model according to Eq. (2) to measured OCT depth scans as described in the previous section. We use three different samples S1, S2, and S3 with different diameters and particle concentrations expressed in weight-% (wt.%) of PS particles in the dispersion, see Fig. 4(d,e) for scanning electron images of the dried particles. The nominal size and concentration of the investigated dispersed particles amount to 143 nm at 2.0 wt.% for S1, 143 nm at 0.5 wt.% for S2, and 246 nm at 0.2 wt.% for S3, respectively. Figure 4(a) depicts averaged OCT depth scans (light coloured circles) along with the curves of the fitted single-scattering model (bright colours). For the fit, measurement data have been taken into account only up to a depth of 1.8 mm (225 measurement points) to avoid inaccuracies by multiple scattering^36^. The extinction coefficient *μ*_t_ and the backscattering coefficient *μ*_b_ can be extracted from the fit: Sample S1 (143 nm, 0.5 wt.%): *μ*_t1_ = (85 ± 23) m^−1^, *μ*_b1_ = (97.0 ± 7.4) × 10^−4^ m^−1^; Sample S2 (143 nm, 2.0 wt.%): *μ*_t2_ = (352 ± 26) m^−1^, *μ*_b2_ = (53.6 ± 5.8) × 10^−3^ m^−1^; Sample S3 (246 nm, 0.2 wt.%): *μ*_t3_ = (125 ± 7.5) m^−1^, *μ*_b3_ = (18.0 ± 2.0) × 10^−3^ m^−1^. The error bounds refer to the standard deviation of the averages. Since the particle concentrations and hence the number densities *N* are known, we can translate these coefficients directly to the scattering cross sections *σ*_s_ and *σ*_b_, assuming that absorption of the PS particles can be neglected at the measurement wavelength around 1315 nm. The following total scattering *σ*_s_ and backscattering cross sections *σ*_b_ have been extracted: Sample S1 (143 nm, 0.5 wt.%): *σ*_s1_ = (25.9 ± 7.0) nm^2^, *σ*_b1_ = (29.7 ± 2.3) × 10^−4^ nm^2^; Sample S2 (143 nm, 2.0 wt.%): *σ*_s2_ = (26.9 ± 2.0) nm^2^, *σ*_b2_ = (41.0 ± 4.4) × 10^−4^ nm^2^; Sample S3 (246 nm, 0.2 wt.%): *σ*_s3_ = (488 ± 29) nm^2^, *σ*_b3_ = (70.1 ± 7.7) × 10^−3^ nm^2^. The values given include the standard deviation of the averages. The cross sections *σ*_s_ and *σ*_b_ can be related to the diameters of the respective particles using Mie’s theory^34^, see Fig. 4(b). The total scattering cross section *σ*_s_ (left axis, red) and the backscattering cross section *σ*_b_ (right axis, blue) of polystyrene nanospheres dispersed in water are calculated and plotted as a function of the sphere diameter, assuming incident light of 1315 nm wavelength and a system aperture of 0.018 (1° half-angle). From the measurement fits in Fig. 4(a), the scattering parameters *σ*_s1_, *σ*_b1_ for sample S1 and *σ*_s3_, *σ*_b3_ for sample S3 are extracted. The sphere diameter can then be read from the Mie calculations displayed in Fig. 4(b), vertical arrows. For clarity, the analysis is only depicted for sample S1, but sample S2 yields very similar results. The table in Fig. 4(c) summarizes the results as derived from scattering cross section *σ*_s_ and backscattering cross section *σ*_b_, together with the associated standard deviation of the averages, and compares them with the nominal particle size and its standard deviation. The standard deviation measured by OCT can be lower than the standard deviation of the particle size distribution, since various particles contribute to one OCT measurement. Measurement errors may arise from refractive index uncertainties, where an uncertainty of 0.01 would lead to a size determination error of 2 nm to 4 nm. Relative deviations between nominally and measured particle sizes are below 4%.

### Model-based nanoscale dispersion-state analysis

Owing to the high sensitivity of the OCT technique, even strongly absorbing nanomaterials such as carbon-nanotube (CNT) composites can be analysed. This is a key feature, as CNT-loaded composites represent an important market segment^37^. Standard light scattering techniques like DLS or SLS are not applicable because the scattered or transmitted optical powers are small. Conventional CNT dispersion analysis relies either on thin material layers that are investigated in a light microscope (LM), or on the determination of macroscopic material parameters like the dielectric permittivity that can also indicate the dispersion quality^38^. For our experiments, we use a multi-wall CNT (MWCNT) dispersion in an epoxy resin (Araldite LY 556, Huntsman Advanced Materials GmbH, Basel, Switzerland). The samples contain 0.12 wt.% of MWCNT (NC7000, Nanocyl S.A., Sambreville, Belgium), and were prepared by 1, 3 and 5 milling cycles in a three-roll mill. We compare the results of OCT-based backscattering and extinction parameter analysis with the results of standard LM analysis as well as with rheological and dielectric measurements. For OCT measurements, the CNT-filled resin samples were filled into cuvettes and heated up to a temperature of 50 °C for melting the resin, thereby avoiding scattering from resin crystals.

**Figure 5:**
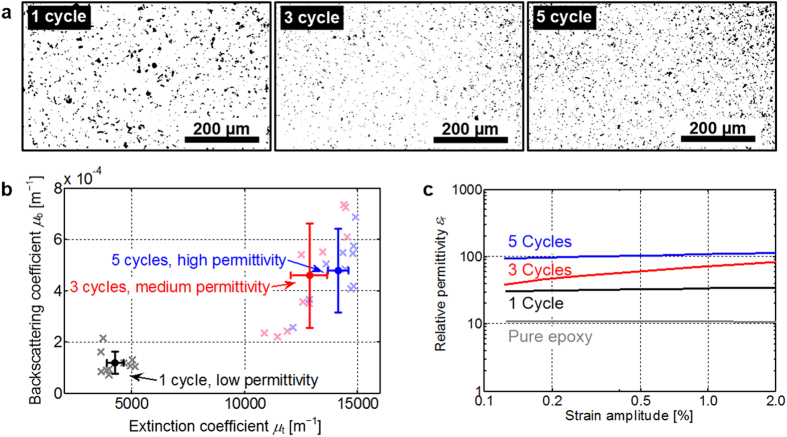
OCT characterization of highly absorbing epoxy/carbon nanotube (CNT) composite materials. Comparison to conventional characterization techniques and macroscopic material properties. The composite was milled over 1, 3 and 5 cycles in a three-roll-mill to improve dispersion. (**a**) Conventional dispersion state analysis by light microscopy of thin composite layers. Black areas are attributed to agglomerates. (**b**) OCT-determined backscattering *μ*_b_ and extinction *μ*_t_ coefficients, measured in 10 different spatial regions (crosses) of each sample (average: solid dots; standard deviations: horizontal and vertical error bars). (**c**) Rheologic dielectric characterization of the relative permittivity ε_r_ as a function of applied shear strain after 1, 3, and 5 milling cycles. The relative permittivity increases monotonically from the pure epoxy material over 1 to 3 to 5 milling cycles, indicating an increase in the separation of the CNT.

The results of the dispersion analysis are shown in Fig. 5. Conventional dispersion analysis of thin composite layers by light microscopy, see Fig. 5(a), reveals significant agglomerates after the first milling cycle. After three milling cycles, the size of agglomerates reduces. After five milling cycles, the light microscope images could lead to the conclusion that dispersion quality seems to decrease again, but other measurement techniques, like OCT analysis and rheological characterization, contradict this finding. Light microscopy for dispersion analysis, while being a state-of-the-art technique, suffers from small imaging areas. This could lead to an erroneous interpretation, if the agglomerate concentration is not spatially homogeneous over the whole sample. The same samples were investigated using OCT, see Fig. 5(b), which shows extinction coefficients *μ*_t_ and the backscattering coefficients *μ*_b_ in a two-dimensional plot. For each of the samples, we take ten measurements, each consisting of 5000 depth-scans which were taken while laterally moving the sample over a distance of 2 mm. The depth scans are averaged, and the extinction coefficient *μ*_t_ and the backscattering coefficient *μ*_b_ are extracted by fitting Eq. (2) to the measurement data. This has been repeated at ten different regions of each sample, each region corresponding to a cross in Fig. 5(b). Note that the particle sizes cannot be extracted from these data: In contrast to the situation for pure nanosphere dispersions used as a reference, the CNT agglomerates exhibit a large variety of shapes, and a Mie scattering theory based on simple spheres cannot be applied. Yet, the analysis of the extinction and backscattering coefficients suffices for a qualitative dispersion analysis. The horizontal and vertical error bars in Fig. 5(b) represent the average (solid dots) and the standard deviation of the measurements obtained for each sample. Although it is not possible to exactly discriminate between scattering and absorption, we may assume that the high extinction values of 5 mm^−1^ up to 15 mm^−1^ arise mainly from absorption of the CNT and only marginally from scattering. This is supported by strong reported absorption^39^ of 1000 mm^−1^ due to separated CNT in composites with comparable concentrations. Consequently, we did not take multi-scattering into account. A clear tendency with respect to the number of milling cycles can be seen: The more agglomerates are broken, the more isolated CNT can contribute to absorption and hence to the extinction *μ*_t_, which rises significantly between one and three milling cycles, and increases slightly for five cycles. Simultaneously, the backscattering coefficient *μ*_b_ increases with the number of milling cycles and shows the same tendency as the extinction *μ*_t_. The backscattering coefficient *μ*_b_ = *σ*_b_ *N*, however, does not necessarily increase if the dispersion quality increases so that the agglomerates become smaller and their number *N* becomes larger. It is impossible to state in general how smaller agglomerates contribute to the backscattering inside the light receiving aperture, because *σ*_b_ strongly depends on the angle dependency of the backscattering for the respective agglomerate shape. In our specific case, *μ*_b_ increases with an increasing degree of dispersion, and therefore allows a qualitative judgement of the state of the sample dispersion.

These results of the OCT analysis are confirmed by a rheological characterization of the relative permittivity ε_r_. For this purpose, the dispersions were investigated using a rheometer (MCR 501, Anton Paar GmbH, Graz, Austria) in combination with a programmable LCR bridge for measuring inductance, capacitance and resistance (HM 8118, HAMEG Instruments GmbH, Mainhausen, Germany). The rheometer consists of two parallel rotatable plates at a separation of *s* = 1 mm. The sample dispersion fills the gap between the plates, which are isolated electrically from each other and which are connected to the LCR bridge for measuring the capacitance. During a measurement, one of the plates performs an oscillating rotation. The amplitude *b* of the oscillation at the outer radius of the plate, related to the plate separation *s* results in the strain amplitude *γ* = *b*/*s*. Both the measurement setup and the measurement procedure are described in detail in ref. 38. The relative permittivity is measured at a frequency of 1 kHz and increases monotonically from the pure epoxy material over 1 to 3 to 5 milling cycles: This indicates a continuous improvement of dispersion quality, see Fig. 5(c), since an increasing number of CNT are separated from agglomerates and contribute to the electric polarization of the nanocomposite. Note that the rheological measurements themselves introduce shear strain into the samples, which could lead to further exfoliation of CNT from the agglomerates and hence to an increase of the relative permittivity during characterization of the samples after three milling cycles. This additional exfoliation might be not possible for the mixture after one milling cycle (insufficient exfoliation) and five milling cycles (largely complete exfoliation). At higher strain amplitudes (not shown), the separated CNT align in parallel to the rheometer plates and thus perpendicularly to the electric field. In this case the CNT cease to influence the permittivity.

In contrast to the microscopy analysis and the permittivity measurement, OCT characterizes the heated sample without any further preparation. The results correlate well with the permittivity measurement and with the number of dispersion cycles. In contrast to light microscopy, OCT also reveals the increase in dispersion quality between the third and the fifth dispersion cycle. This experiment demonstrates that OCT metrology is useful to characterize even highly absorbing nanomaterials, outperforming by far the elaborate and time consuming conventional light microscopy method.

### Image-based dispersion-state analysis for microscale agglomerates

**Figure 6:**
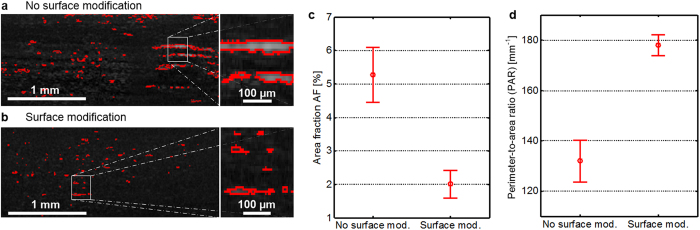
Image-based dispersion-state analysis for microscale agglomerates, showing the impact of chemical surface modification on dispersibility. (**a**) Cross-sectional image (B-Scan) of a PA/nanoclay composite with 5 wt-% nanoclay content without surface modification. Bright spots (strong backscattering) with red borders indicate agglomerates. Right part: magnified section. (**b**) Cross-sectional image of a PA/nanoclay-composite with surface-modified clay particles having the same concentration as the sample in (**a**). Right part: magnified section. (**c**) Area fraction (AF) covered by detected agglomerates. The circles denote average values of the three samples, and the error bars indicate the standard deviation of the average. (**d**) Perimeter-to-area ratio (PAR) of the detected agglomerates. The circles denote average values of the three samples, and the error bars indicate the standard deviation of the average.

For experimentally assessing the ability of the OCT technique to analyse nanocomposites with large agglomerates, composites of polyamide (PA; Badamid B70, Bada AG, Bühl, Germany) and nanoclay particles with 5 wt.% concentration were prepared in a compounding extruder^40^. For controlling the dispersion quality during fabrication, one batch of samples was prepared from a clay with modified surface (I.34, Nanocor Inc., Hoffman Estates, IL, USA), the other one was prepared from unmodified clay (PGN, Nanocor Inc.). The surface modification is expected to lead to an improved dispersion state and to decreased sizes of agglomerates as compared to the unmodified clay. After compounding, all samples are granulated without further sample processing. Cross-sectional images (B-scans) are taken from the granules with our laboratory OCT system. Representative B-scans from the PA/nanoclay composite without and with surface modification are shown in Fig. 6(a,b), where the pixel brightness indicates measured backscattering. The sample with no modification, Fig. 6(a), shows large lengthy bright areas, indicating large clay agglomerates that extend over several hundreds of micrometres, whereas the sample with modification, Fig. 6(b), features small bright spots only from which small agglomerates can be inferred. The image segmentation algorithm detects bright regions automatically; the corresponding borders are drawn as red lines.

For each sample type, three B-scans are taken, segmented, and the area fraction (AF) as well as the perimeter-to-area ratio (PAR) are calculated for each measurement. Figure 6(c,d) display the average AF and the average PAR for both sample types along with the corresponding error bars showing the standard deviation of the averages. A significant decrease in the AF is obtained when the surface of the clay filler is modified (AF_mod_ = 2.0% ± 0.4%) as compared to the filler without surface modifications (AF_unmod_ = 5.3% ± 0.8%), Fig. 6(c). This indicates that a surface modification significantly decreases the agglomerate size such that a substantial fraction of agglomerates becomes smaller than the size detection threshold of the image analysis. This is in accordance with the observation that the cross-sectional image of the surface-modified clay composite exhibits a large number of bright spots, each of which contains only a few pixels, see Fig. 6(b). At the same time, the PAR increases significantly with modification of the filler surface (PAR_unmod_ = 132 mm^−1^ ± 8.2 mm^−1^, PAR_mod_ = 178 mm^−1^ ± 4.1 mm^−1^) which confirms the decrease in average agglomerate size, Fig. 6(d). These experiments prove the viability of image-based OCT analysis to characterize the dispersion state of samples with relatively large agglomerates sized from a few micrometres to hundreds of micrometres. The quantitative evaluation of further dispersion-related parameters like agglomerate shape and number would further increase the reliability and robustness of our technique.

### Demonstration of in-line dispersion-state analysis

Nanocomposite development is hampered by rather long development cycles, which are dominated by time consuming off-line characterization. In this section, OCT is demonstrated to be well-suited for continuous dispersion-state monitoring in a production environment.

**Figure 7:**
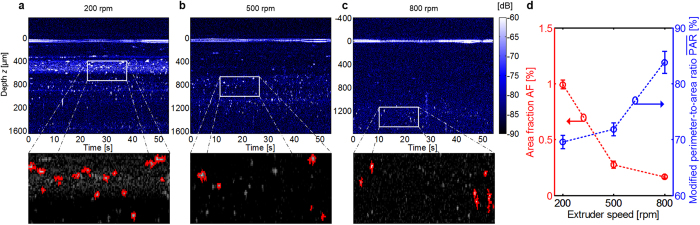
Demonstration of in-line dispersion-state analysis during operation of a compounding extruder. The OCT measurement beam is fixed, the vertical axis of the OCT images indicates the imaging depth and the horizontal axis corresponds to the measurement time. Bright spots: strong backscatter of agglomerates. Straight horizontal line at *z* ≈ 0: permanent reflections at the probe window. Material system: polypropylene/nanoclay. Mass flow: 6 kg/h. Clay content: 1%. Bottom: magnification after segmentation. (**a**) OCT scan at 200 rpm. (**b**) OCT scan at 500 rpm. (**c**) OCT scan at 800 rpm. (**d**) Quantitative analysis of the dispersion state by area fraction (AF) and modified perimeter-to-area ratio 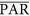 from five OCT scans. The circles denote average values of the five scans, and the error bars indicate the standard deviation of the averages.

In the following experiment, a production-scale twin-screw compounding extruder (Leistritz GmbH, Nuremberg, Germany) is used for production of a polypropylene(PP)/nanoclay composite with a mass throughput of 6 kg/h. The applied host material is PP (R352-08R, Dow Chemical, Midland, MI, USA) and the nanosized filler is a nanoclay (Cloisite 15A, Southern Clay Products, Gonzales, TX, USA). The screws introduce shear into the polymer melt filled with nanoparticles and thus disperse the particles^40^. With this extruder, nanocomposites were dispersed with different revolution speeds. In general, increasing speed comes with higher energy input into the material and causes a better nanoparticle dispersion^12^. In order to characterize the dispersion state of the PP/clay melt during production, the extruder has been equipped with the OCT probe for in-line process monitoring, see Fig. 1(c). The probe is mounted to a sensor port of the extruder located at the end of the machine close to the die. The optical window of the probe is in direct contact with the main stream of the extruded nanoparticle loaded polymer melt. With the OCT probe, A-scans of the medium underneath the probe window are measured continuously. The flow of the melt causes a continuous movement of the material seen by the OCT measurement beam, thereby replacing a lateral movement of the measurement beam in conventional measurements. This results in a temporal change of the backscatter signal, which can be interpreted as 2D image data, where one dimension corresponds to time and the other dimension is the usual imaging depth measured from the probe window, see Fig. 7.

Figure 7(a–c) show the in-line OCT data for the screw revolution speeds 200 rpm, 500 rpm and 800 rpm, respectively. Note that, due to non-uniform mass flow as a function of depth, it not possible to directly map the measurement time (horizontal axis) to spatial coordinates of the sample. Bright spots indicate strong backscatter and are attributed to agglomerates. The straight horizontal line at *z* ≈ 0 originates from permanent reflections at the probe window. With higher screw speed and accordingly higher shear strain inside the material, fewer agglomerates are visible indicating an improvement in dispersion quality. At the highest screw speed of 800 rpm, almost no agglomerates are visible. This impression is also confirmed by quantitative dispersion-related parameters. Since the measurement is performed as a function of time rather than as a function of lateral position, a perimeter-to-area ratio in the strict sense as defined by Eq. (4) cannot be calculated. Instead, we use a modified quantity PAR, which essentially corresponds to the PAR except that both, the agglomerate perimeters *s*_i_ and the agglomerate areas *A*_i_, are expressed by pixel numbers rather than by physical lengths and areas. Note that PAR. a dimensionless quantity in contrast to PAR, which has the unit m^−1^. The results are depicted in Fig. 7(d), where the circles denote average values from five OCT scans, and the error bars denote the respective standard deviations of the averages. With increasing speed, the area fraction AF reduces, and the PAR rises. Both quantities indicate that the agglomerates inside the material flow become smaller corresponding to a better dispersion quality of the composite. These results reveal a clear relationship between operation parameters of the machine and OCT-measured dispersion parameters. This is the first demonstration of an in-line dispersion-state analysis in a nanocomposite production line. The results encourage the application of our technique to more material systems for controlling multiple production parameters.

## Conclusions

In this work, we present a novel and a highly attractive approach to nanomaterial analysis applied to nanoparticles and nanocomposites. The method uses optical coherence tomography (OCT) and model-based parameter extraction. Our approach enables detection and quantification of nanoparticles and agglomerates over a wide range of size scales: Image segmentation of OCT data sets is well suited for dispersion-state characterization of nanocomposites with agglomerates in the micrometre range, whereas model-based scattering analysis lends itself to size determination of nanoparticles below the resolution limit. We elaborate the measurement technique along with theoretical models and demonstrate the viability of the procedure in a series of proof-of-principle experiments. A wide variety of material systems is investigated by our experiments: A first demonstration shows accurate OCT-based nanoparticle sizing. An *in-situ* dispersion-state analysis characterizes strongly absorbing CNT composites in liquid media. Finally, we perform in-line monitoring of the compounding process in a state-of-the-art production line. Major challenges in industrial applications are the stringent requirements with respect to mechanical stability and size of the OCT system. These requirements can be met by integration of interferometer and detection system on a silicon photonic chip^41^. We believe that OCT has the potential to fill a metrology gap in the emerging field of nanocomposite technology. We conclude that OCT metrology opens new directions in material analysis, both in laboratory and production environments.

## Additional Information

**How to cite this article**: Schneider, S. *et al*. Multiscale dispersion-state characterization of nanocomposites using optical coherence tomography. *Sci. Rep.* **6**, 31733; doi: 10.1038/srep31733 (2016).
